# Recovery of Theta Frequency Oscillations in Rats Following Lateral Fluid Percussion Corresponds With a Mild Cognitive Phenotype

**DOI:** 10.3389/fneur.2020.600171

**Published:** 2020-12-04

**Authors:** Katelynn Ondek, Aleksandr Pevzner, Kayleen Tercovich, Amber M. Schedlbauer, Ali Izadi, Arne D. Ekstrom, Stephen L. Cowen, Kiarash Shahlaie, Gene G. Gurkoff

**Affiliations:** ^1^Department of Neurological Surgery, University of California, Davis, Davis, CA, United States; ^2^Center for Neuroscience, University of California, Davis, Davis, CA, United States; ^3^Department of Psychology, The University of Arizona, Tucson, AZ, United States; ^4^McKnight Brain Institute, The University of Arizona, Tucson, AZ, United States

**Keywords:** spatial learning, traumatic brain injury, theta oscillations, biomarker, *in vivo* electrophysiology, phase coherence

## Abstract

Whether from a fall, sports concussion, or even combat injury, there is a critical need to identify when an individual is able to return to play or work following traumatic brain injury (TBI). Electroencephalogram (EEG) and local field potentials (LFP) represent potential tools to monitor circuit-level abnormalities related to learning and memory: specifically, theta oscillations can be readily observed and play a critical role in cognition. Following moderate traumatic brain injury in the rat, lasting changes in theta oscillations coincide with deficits in spatial learning. We hypothesized, therefore, that theta oscillations can be used as an objective biomarker of recovery, with a return of oscillatory activity corresponding with improved spatial learning. In the current study, LFP were recorded from dorsal hippocampus and anterior cingulate in awake, behaving adult Sprague Dawley rats in both a novel environment on post-injury days 3 and 7, and Barnes maze spatial navigation on post-injury days 8–11. Theta oscillations, as measured by power, theta-delta ratio, peak theta frequency, and phase coherence, were significantly altered on day 3, but had largely recovered by day 7 post-injury. Injured rats had a mild behavioral phenotype and were not different from shams on the Barnes maze, as measured by escape latency. Injured rats did use suboptimal search strategies. Combined with our previous findings that demonstrated a correlation between persistent alterations in theta oscillations and spatial learning deficits, these new data suggest that neural oscillations, and particularly theta oscillations, have potential as a biomarker to monitor recovery of brain function following TBI. Specifically, we now demonstrate that oscillations are depressed following injury, but as oscillations recover, so does behavior.

## Introduction

At least 3.8 million traumatic brain injuries (TBI) occur each year (1, 2), costing an estimated $221 billion annually, with ~95% of that cost attributed to long-term care (3). While chronic disability is typically associated with moderate and severe TBI, there is considerable evidence that a subset of injuries initially diagnosed as mild (mTBI) also results in persistent cognitive and affective dysfunction (4–6) that may last months to years (7–9), even in the absence of clinically-identifiable anatomical or motor deficits (10–12). While the majority of TBI are more mild, there is evidence that patients have a window of vulnerability to a subsequent injury (13–16), yet we currently do not have an objective diagnostic tool based on known mechanistic changes that can determine when a patient has sufficiently recovered. Identifying sensitive and affordable biological markers of recovery is critical, as individuals who experience repeat traumatic brain injury are more likely to experience lasting disability (14, 17–23).

One potential electrophysiological biomarker for altered neural activity and recovery of function is the theta rhythm. Theta is a large, slow wave oscillation (5–12 Hz) that can be measured in the non-invasive electroencephalogram (EEG) and also recordings of local field potentials (LFP) from depth electrodes implanted in brain regions such as the hippocampus (24–27). Hippocampal theta oscillations synchronize activity both within local networks and across distal cortical regions involved in cognitive processing (28, 29). In both humans and rats, theta power increases during the acquisition phase of spatial and object-based learning tasks (30–32). In rodents, theta oscillations predominate during periods of exploration and sensory processing (24, 33), with hippocampal place cells firing in relative phase with the theta rhythm, a phenomenon called phase precession (34–38). Moreover, high-powered theta oscillations are predictive of successful performance on cognitive spatial tasks and, in particular, spatial learning (39, 40). Chemical- (41, 42) or injury-induced (43, 44) inhibition of theta oscillations leads to cognitive dysfunction, and cross frequency relationships with gamma (28–64 Hz) waves are implicated in memory encoding and recall (45).

Previously, we and others have demonstrated that neural oscillations were disrupted in rodent models of TBI (43, 46–48). Building on these previous results, we hypothesized that a single TBI would result in significant changes in the septohippocampocortical network connectivity, including attenuated theta power and altered phase coherence. Moreover, we predicted that altered oscillatory activity would correlate with behavioral deficits in spatial memory. The ability to detect oscillatory changes that link to behavioral deficits would represent a significant advancement for identifying markers of injury and informing return-to-function decisions. Moreover, if findings from implantable depth electrodes can ultimately be observed in extracranial EEG, then oscillatory activity could be used as an objective, fast, inexpensive, and non-invasive measure related to recovery of function following TBI.

## Materials and Methods

### Animals and Groups

All experiments involving animals complied with the ARRIVE guidelines and were performed in accordance with the National Institutes of Health guide for the care and use of laboratory animals (NIH publication No. 8023, revised 1978), following procedures approved by the University of California, Davis Institutional Animal Care and Use Committee (Protocol #19612).

### Housing, Husbandry, and Humane Endpoints

Adult male Sprague-Dawley rats (*n* = 65; 300–375 g; Envigo, Livermore, CA, USA) were housed in a standard institutional vivarium with a 12-h (7 a.m. to 7 p.m.) light cycle. Following implant, animals were housed individually in acrylic cages with corn cob bedding and *ad libitum* access to water and adult laboratory rodent chow. Bags of crinkle paper were provided for environmental enrichment and changed weekly. Room conditions were monitored daily to maintain temperature of 68–79°F and humidity of 30–70%, and fresh food and water were provided weekly. Welfare assessments were conducted daily by laboratory staff, including weight checks and provision of parenteral nutrition, including subcutaneous injection of lactated Ringer's solution (Hospira, Inc, Lake Forest, IL) and/or provision of Supplical High Calorie Veterinary Supplement (Henry and Schein, Inc, Melville, NY), as needed. Animals with a sustained weight loss greater than or equal to 20% were removed from the study and euthanized humanely, according to the IACUC Protocol. All procedures adhere to the National Institutes of Health guidelines and were approved by the University of California, Davis Institutional Animal Care and Use Committee.

### Experimental Animals and Group Sizes

Prior to surgery, the cage identification cards were shuffled and blindly drawn to randomly assign animals to sham control (*n* = 15) and lateral fluid percussion injury (*n* = 50) groups. Nine animals from the injury group either died during recovery or were euthanized due to complications in the week following injury.

### Lateral Fluid Percussion

Lateral fluid percussion injury was delivered as previously described (49). Sham animals received the same surgical procedures as TBI except the fluid percussion injury was not administered. Anesthesia was induced in a small Plexiglas box using 4% isoflurane (in air). Animals were then intubated, shaved, transferred to a stereotaxic frame, and mechanically ventilated to maintain a surgical plane of anesthesia using 1.5–3% isoflurane (in 2 NO_2_: 1 O_2_) for the remainder of the surgery. After sterile preparation (3x alternating with 70% ethanol and betadine solution), a subcutaneous injection of 0.25% bupivacaine (0.1 mL) was delivered to the shaved skin on the dorsal surface of the skull. A midline scalp incision was made, and the skin was retracted to expose the dorsal cranial surface. Viscous dental etchant (37% phosphoric acid) was applied directly to the skull for ~60 s to increase surface area for the subsequent implant, and then the skull was flushed with sterile saline. Using a trephine, a circular 4.8 mm diameter parasagittal craniectomy was centered 3 mm posterior to bregma and 3 mm lateral to midline over the right hemisphere. Two stainless steel screws (0–80″) were secured in the skull, anterior and posterior to the craniectomy. A plastic tube injury hub (custom, made in-house) was placed in the craniectomy and cemented to the skull with a combination of super glue gel and dental acrylic, which was anchored to the support screws. The injury hub was then filled with sterile saline.

The fluid percussion device was calibrated to produce an injury of ~2.1 atm of pressure. In our lab, this injury typically results in persistent spatial learning deficits for at least 2 weeks post-injury (46, 50). The animal was removed from anesthesia, and the fluid percussion device was used to impinge a small quantity of sterile saline onto the surface of the dura, resulting in a mean pressure of 2.12 ± 0.033 (atm ± SD). Immediately following injury, animals were returned to 2.5% isoflurane and, after a surgical plane of anesthesia was re-established, electrodes were implanted.

### Electrode Implantation

**Figure 1:**
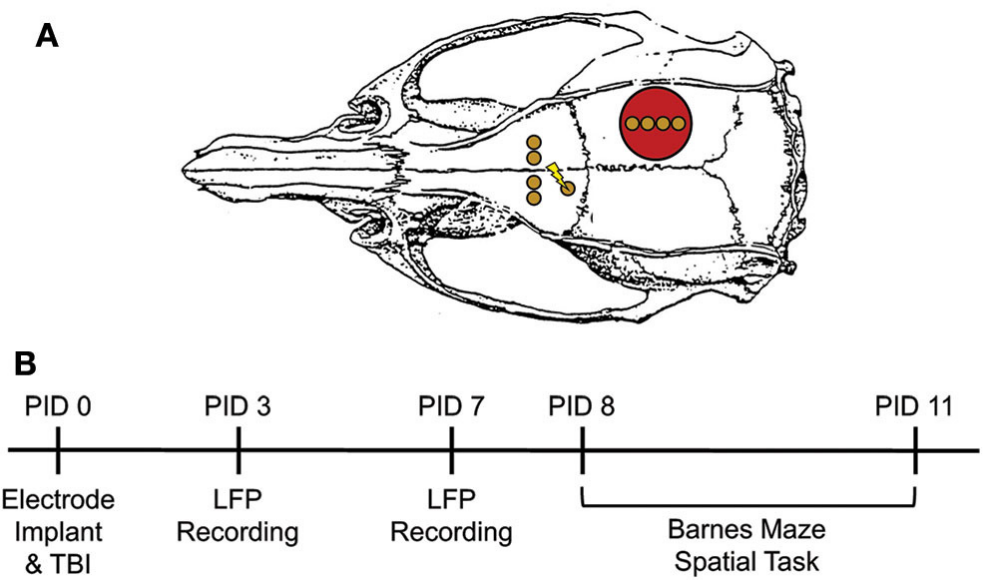
Experimental Design. **(A)** Electrode configuration and craniotomy location for all animals. Red circle = site of craniotomy/injury. Gold circle = electrode implantation site. Lightning bolt = stimulating electrode pair. **(B)** Experimental Timeline. Electrodes were immediately implanted following FPI or sham injury or PID0. Animals were placed in a novel context on PID3 and 7 for LFP recordings. On PID8-11, animals underwent testing on the Barnes maze. This consisted of two 3-min trials per day with a 5 min ITI. Pre-task LFP was recorded in a small box prior to trial 1 each day. Adapted from Paxinos et al. (51).

Using a stereotaxic arm (Model 940, David Kopf Instruments, Tujunga, CA, USA), an array of 4 tungsten electrodes (E363T/2/SPC, 200 μm diameter, 17–23 kΩ impedance; Plastics One Inc., Roanoke, VA, USA) was staggered along the ML and DV axes in the ipsilateral CA1 of the dorsal hippocampus (dHPC; AP: −4.5 mm, ML: +3.1 to +3.6 mm, DV: _−2.8 to −3.1 mm) (Figure 1A). The shortest electrode was the most medial, and each subsequent electrode was at a similar AP coordinate but ~125 μm more lateral and ~100 μm more ventral, in order to account for the shape of the dHPC. A second array was implanted in the anterior cingulate cortex (ACC) and was staggered along the AP and DV axes (AP: +1.7 mm to +2.2 mm, ML: + 0.40 mm, DV: −4.00 to −3.5 mm). The shortest electrode was the most anterior, and each subsequent electrode was at a similar ML coordinate but ~125 μm more posterior and ~100 μm more ventral. A pair of tungsten electrodes was lowered into the medial septal nucleus (MSN; AP: +0.48 mm, ML: −1.52 mm, DV: −7.01 mm, 10° angle) for bipolar stimulation. All electrodes were connected to an electrode interface board (EIB-Q-16, Neuralynx, Bozeman, MT, USA) using stainless-steel wire. A cerebellar screw served as a ground/reference. A combination of stainless steel screws (0–80″), super glue gel, C & B MetaBond Quick Adhesive Cement System (Parkell, Edgewood, NY, USA), and dental acrylic were used to create a stable implant. Animals were then removed from anesthesia and monitored during recovery.

### Handling, Habituation, and Novel Context Recordings

Rats were handled for ~5 min/day for 5 days pre-surgery and again daily post-surgery (see Figure 1B, for timeline). Oscillatory activity was measured on post-injury day 3 (PID3) and 7 while animals freely explored a small open field (29.2 × 29.2 × 27.9 cm) for 10 min. LFP were recorded via a head stage preamplifier (HS-16-QC, Neuralynx, Bozeman, MT, USA) tethered to a motorized commutator and ultimately to a 16 channel Neuralynx Digital Lynx acquisition system (Digital Lynx SX; band-pass filtered 0.1–2,000 Hz, 32 kHz sampling rate). All channels were referenced to the cerebellar screw electrode, which also served as the ground. Oscillatory activity was evaluated by two investigators who were blind to group.

### Barnes Maze Task

The Barnes maze apparatus is a black matte circular platform (146 cm diameter) with 22 circular holes (14 cm diameter) equally spaced along the periphery. A black bath curtain was hung ~0.5 m from the edge of the maze and four distinct cues were spaced equally around the curtain and in the rat's visual field. A black escape box was fixed under one of the circular holes not quite centered between two of the cues. A cable tray was mounted ~1.5 m directly above the apparatus. Two 400-watt LED lights (aimed toward the ceiling) were positioned to generate even illumination, and a 50 dB white noise generator was centered above the maze. The lights and noise generator were each connected to a Bluetooth-controlled power strip, with a switch mounted on the wall next to the maze.

On PID7, animals were habituated to the maze. Specifically, cues were removed from the curtain, the goal box was removed, and lights were set to a low level. Animals were placed in the middle of the maze and given 5 min to explore. Starting on PID8, animals received two trials a day for 4 consecutive days, totaling eight trials. All trials were conducted in similar environmental conditions, including room temperature and humidity, lighting level, and time of day. Prior to the first trial each day, animals were attached to a tether and placed in a small black box outside the maze for a 10-min baseline recording. LFP from these pre-trial recordings was compared to recordings from the Novel Context, as the rats were in a similarly constrained space. Rats were then transferred to a start box centered on the maze. After 15 s, the start box was lifted to expose the animal to the apparatus, and the white noise generator and two 400-watt LED lights were turned on for the duration of the trial. Each trial was 3 min long or until the rat entered the escape box. Animals that did not find the escape box in 3 min were guided to it. After 1 min in the escape box, rats were placed back into the small black box for 4 min, resulting in a total inter-trial interval of 5 min. After completion of the 2nd trial, animals were transferred back to the small black box for 10 additional min of recording prior to being returned to their home cage. All trials were video recorded and manually scored by a blind observer for latency to find the hidden escape box, search strategy, and number of errors. Strategy was categorized as “direct” if that animal oriented toward the escape box and directly investigated within 2 holes of the correct entry point. If an animal went to the edge of the arena and searched holes in a serial fashion, skipping no more than 2 holes, it was scored as a “peripheral” strategy. “Random” strategies were assigned when the animal skipped 3 or more holes while searching and did not appear to use a direct or peripheral approach. As trial 1 is necessarily random„ only trials 2–8 were compared for both latency and search strategy.

### Stimulation Paradigms

Stimulation of the medial septal nucleus occurred exclusively during Barnes maze testing (PID8–11). A twisted wire cable connected the implanted stimulating electrodes to an isolated pulse stimulator (model 2100, A-M Systems, Sequim, WA, USA). Stimulation lasted for exactly 20 min, beginning 8 min before the animal was placed in the start box, continuing through both trials and into the post-Barnes recording period. This stimulation paradigm ensured that all animals received similar durations of stimulation prior to and throughout the Barnes maze training. As latency to find the escape box was variable between animals, there was a difference in time animals were stimulated post-Barnes maze.

**Figure 2:**
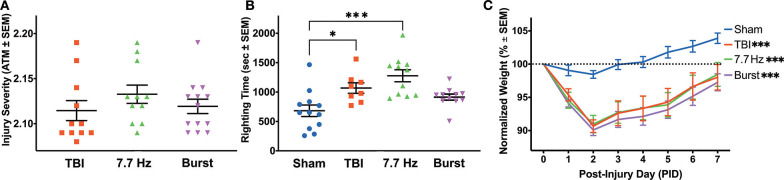
Injured Animals had Impaired Recovery from Surgery, Regardless of Treatment Group. **(A)** Peak atmospheric pressure was used as a measure of injury severity to counterbalance TBI animals into treatment groups. **(B)** There were significant differences between groups in righting time following surgery. The TBI and 7.7 Hz groups had significantly elevated righting times compared to sham (Sham vs. TBI: *p* < 0.05; Sham vs. 7.7 Hz: *p* < 0.001). **(C)** All injury groups had a significantly larger weight loss following injury compared to sham (*p* < 0.001), and there were effects of group and time, as well as an interaction between the two, on recovery in the week following surgery. All data are presented as mean ± SEM, ^*^p < 0.05, ^***^p < 0.001. Adapted from Paxinos et al. (51).

On PID8, injury severity (atm) was used to counter-balance TBI animals into non-stimulated (*n* = 12 “TBI”), continuous 7.7 Hz stimulation (*n* = 11 “7.7 Hz”), and theta burst stimulation (*n* = 13 “Burst”) treatment groups, such that there were no significant differences between groups [Figure 2A, F_(2, 32)_ = 0.9146]. The 7.7 Hz paradigm consisted of continuous, square-wave stimulation at 7.7 Hz, with a current of 80 μA and a pulse-width of 1 ms (42, 47, 52, 53). The theta burst paradigm consisted of 50 ms trains of 200 Hz, 5 trains per second, at 60 μA and 100 μs pulse-width (54).

### Local Field Potential Analyses

Recordings were visually inspected, and artifacts (e.g., disconnections, stimulation artifact) were manually rejected from all subsequent analyses. Raw data were then downsampled to 1,000 Hz and filtered for 60 Hz line noise using a Butterworth band stop IIR filter. LFP were analyzed with custom MATLAB (MathWorks Inc, Natick, MA, USA) scripts developed in-house for (1) oscillatory power, (2) mean peak theta frequency, and (3) phase coherence between the two dHPC electrodes with the highest power, as well as between the highest-powered dHPC electrode and one ACC electrode. Theta was operationally defined as 6–10 Hz (43, 55), and spectral power estimates across frequency bands (2–30 Hz, 30 logarithmically spaced points) were calculated using Morlet wavelets [cycles = 6; (56)] and normalized to decibels using the ratio of power in the first minute to the last minute within each 5-min recording session. In the HPC, oscillatory power varies significantly along the dorsal-ventral axis with the highest amplitude oscillations occurring proximal to the hippocampal fissure (33). Therefore, for all LFP analyses in the dHPC, the electrode with the highest theta power on PID7 was selected for all subsequent analyses, with exception of intra-hippocampal coherence, for which the phase relationship was compared between electrodes with the highest and second-highest power. As oscillations were of similar frequency and amplitude within the ACC, each electrode was analyzed, and an average value was generated for final statistical analyses. Animals were excluded from electrophysiological analyses if recordings were corrupted or if quality was not adequate for analysis in either the novel context (*n* = 8 sham and *n* = 6 TBI excluded) or Barnes maze (*n* = 2 sham, *n* = 3 TBI excluded). In addition, due to stimulation artifact, only sham and unstimulated animals were included in electrophysiological analyses during the Barnes maze task.

### Statistics

Graphpad Prism statistical software version 8.3 (GraphPad Software, San Diego, CA, USA) was used for all analyses. A one-way ANOVA was used to compare the magnitude of fluid percussion (atm) between stimulated and unstimulated injured animals. Differences in righting time following injury and electrode implantation surgery were also evaluated across all four groups using a one-way ANOVA, with a Dunnett *post-hoc* test comparing each injury group to sham controls. A repeated measure analysis of variance (rmANOVA) was used to compare effects of injury and time on weight change following surgery, with a Bonferroni *post-hoc* analysis.

Prior to stimulation on day 8, all injured animals were treated identically and analyzed as a single injury group. For PID3 and 7 analyses, a three-way ANOVA was used to compare effects of injury and frequency across time post-injury on theta power in each region. A fixed effects type III model was used to compare the effects of injury and time on (1) mean peak theta frequency and (2) theta-delta ratio (TDR), with Bonferroni *post-hoc* tests where applicable. A Wilcoxon signed rank test was used to analyze coherence measures, using the median sham coherence, averaged in the time domain, as the test condition. As certain animals were missing data points on the Barnes maze, a mixed effects model was used to analyze the effects of treatment group and time on latency to find the escape box during Barnes maze training. Chi-square tests were used to compare search strategies of all groups to untreated TBI controls. Multiple *t-*tests with a Bonferroni correction for multiple analyses was used to analyze the effect of group at frequencies within the theta range on the power spectral density (PSD) in each region on PSD11 for sham and unstimulated injured rats. As these recordings represent a different behavioral paradigm than the Novel Context recordings on PID3 and 7, and as only unstimulated injured rats are included in the analysis due to stimulation artifact, these data were analyzed separately from the PID3 and 7 recordings.

## Results

### Acute Measures of Injury

As measured by the in-line transducer on the fluid percussion device, there was no significant difference in injury severity between the three injury groups [Figure 2A; F_(2, 32)_ = 0.9]. Neither 7.7 Hz (2.133 ± 0.010 atm, *p* = 0.33) nor Burst (2.119 ± 0.008 atm, *p* = 0.91) groups had statistically different mean injuries than TBI (2.115 ± 0.011 atm). Unlike traditional assessments following lateral fluid percussion, in which righting time is assessed immediately following injury, electrodes were implanted prior to recovering each animal from anesthesia. Even with a significant delay between injury and evaluation, there was a significant effect of group on righting time [F_(3, 38)_ = 8.618, *p* < 0.001], due to both the TBI (1068.3 ± 89.4 s, *p* < 0.05) and 7.7 Hz (1276.6 ± 102.0 s, *p* < 0.001) groups having significantly longer recovery times compared to sham (681.7 ± 98.0 s, Figure 2B). Righting times in the Burst group were not significantly increased (914.0 ± 51.7 s, *p* = 0.15) from sham levels. Injured animals also had significantly different weight recovery than shams. Specifically, there were main effects of time [F_(1.879, 78.93)_ = 59.20, *p* < 0.001] and group [F_(3, 42)_ = 9.204, *p* < 0.001], as well as an interaction between time and group [F_(21, 294)_ = 4.674, *p* < 0.001] when analyzing weight change from baseline in the 7 days following surgery (Figure 2C). Bonferoni *post-hoc* analysis revealed that all injury groups were significantly different from sham (*p* < 0.001), but there were no differences between any of the injured groups.

### Oscillatory Aberrations in the Septohippocampal Circuit Following Injury

To evaluate the disruption of septohippocampal oscillations as a potential mechanism of cognitive deficit following fluid percussion, we analyzed LFP from the dHPC and ACC in a novel context on PID3 and PID7. As previously stated, prior to stimulation, comparisons were between sham and all injured animals. In both regions, we found that injury significantly attenuated theta oscillations across multiple measures acutely and that these aberrations resolved over time.

### Hippocampus

**Figure 3:**
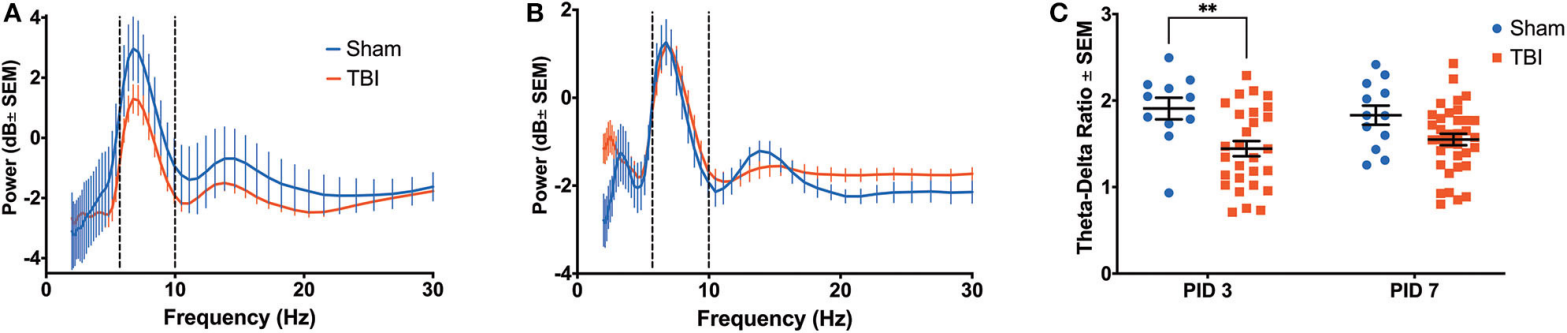
Hippocampal Recovery Following Injury. A three-way ANOVA identified a significant effect of power (*p* < 0.001), time (*p* < 0.001) and group (*p* < 0.001) on theta oscillatory power over the first week post-injury. Dashed lines highlight the theta range (6–10 Hz). **(A)** A power spectral density from the hippocampus on PID3 reveals lower theta power in injured animals compared to shams. **(B)** By PID7, hippocampal theta power had returned to sham-like levels. **(C)** There was a primary effect of group on TDR over time (*p* < 0.01), but *post-hoc* analysis revealed that the two groups only significantly differed on PID3. All data are presented as mean ± SEM, ^**^p < 0.01. Adapted from Paxinos et al. (51).

A three-way ANOVA of hippocampal theta power revealed significant main effects of frequency [F_(9, 820)_ = 19.08, *p* < 0.001], time [F_(1, 820)_ = 18.80, *p* < 0.001], and group [F_(1, 820)_ = 13.26, *p* < 0.001], as well as an interaction between time and group [F_(1, 820)_ = 20.54, *p* < 0.001]. Injured animals had decreased hippocampal theta power on PID3 (0.0716 ± 0.4163 dB; Figure 3A) compared to Sham (1.4699 ± 0.9954 dB), but average theta power (0.1053 ± 0.3267 dB) recovered to sham-like levels (−0.0471 ± 0.4556 dB) by PID7 (Figure 3B). Given the broadband changes reported in other models of TBI (48), we also looked at a separate 3-way ANOVA of hippocampal delta (2–4 Hz) power, which indicated a main effect of time [F_(1, 1, 079)_ = 61.39, *p* < 0.001] and a trend toward a difference between groups [F_(1, 1, 079)_ = 3.077, *p* = 0.080]. We calculated the ratio of theta to delta power (TDR) as a metric of broadband changes. Specifically, an unchanged TDR would indicate a general reduction in total oscillatory power, as theta and delta power would be similarly attenuated. Analysis revealed a primary effect of group [F_(1, 46)_ = 10.49; *p* < 0.01] on TDR (Figure 3C), suggesting a significant drop in theta power relative to delta. The difference in TDR was significant on PID3 (Sham = 1.908 ± 0.125; TBI = 1.444 ± 0.088; *p* < 0.01, df = 82) but had resolved by PID7 (Sham = 1.830 ± 0.111; TBI = 1.550 ± 0.066; *p* = 0.10). There were no differences between groups in the frequency with the highest, or peak, theta power (data not shown; F_(1, 46)_ = 0.2637; *p* = 0.61).

**Figure 4:**
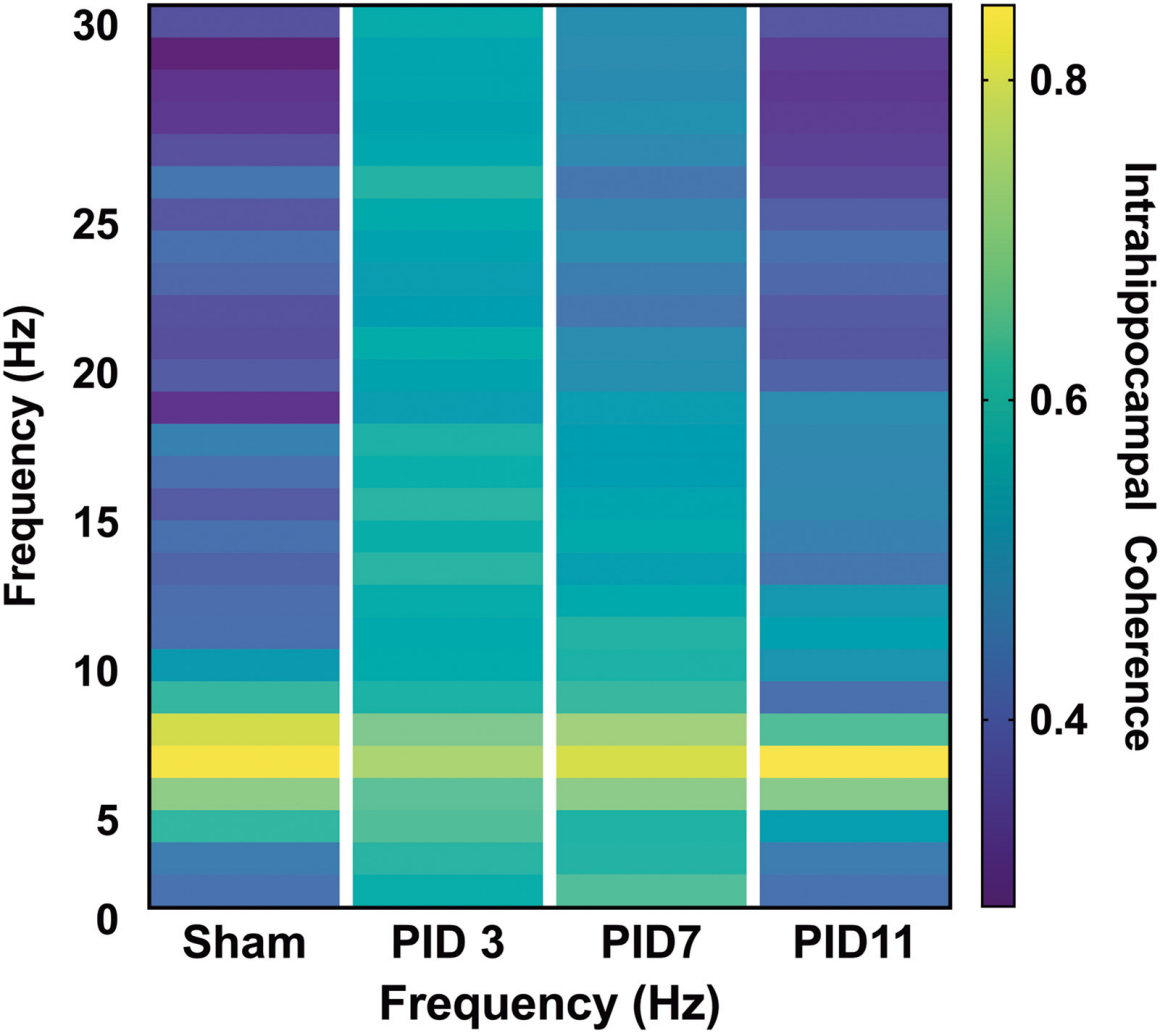
Intrahippocampal Phase Coherence. **(A)** In sham animals, high phase coherence was observed between two neighboring hippocampal electrodes specifically in the theta-frequency range. **(B)** While there was not a significant decrease in theta phase coherence 3 days post-injury (*p* = 0.056), there was a significant increase in coherence in the beta band (*p* < 0.001). **(C)** At 7 days post-injury beta frequency coherence remained elevated (*p* < 0.01) but **(D)** had returned to sham levels by post-injury day 11. Dashed lines highlight the theta band (6–10 Hz). Adapted from Paxinos et al. (51).

There was clear evidence of phase coherence between the top-powered hippocampal electrode and its adjacent electrode in sham animals (Figure 4A). On PID3, injured animals showed decreased coherence in the theta band (Figure 4B; Sham median = 0.8930; TBI median = 0.8341; *p* < 0.05), but a significant increase in the beta frequency band (13–30 Hz), according to a Wilcoxon signed rank test to the Sham median (Sham median = 0.3563; TBI median = 0.6253; *p* < 0.001). Intrahippocampal theta remained significantly decreased on PID7 (Sham median = 0.8874; TBI median = 0.8608; *p* < 0.05), but beta coherence had recovered to sham-like levels (Figure 4C; Sham median = 0.4439; TBI median = 0.4461; *p* = 0.37). By PID11, theta phase coherence had returned to sham-like levels (Figure 4D; Sham median = 0.6596; TBI median = 0.7600; *p* = 0.65).

### ACC

**Figure 5:**
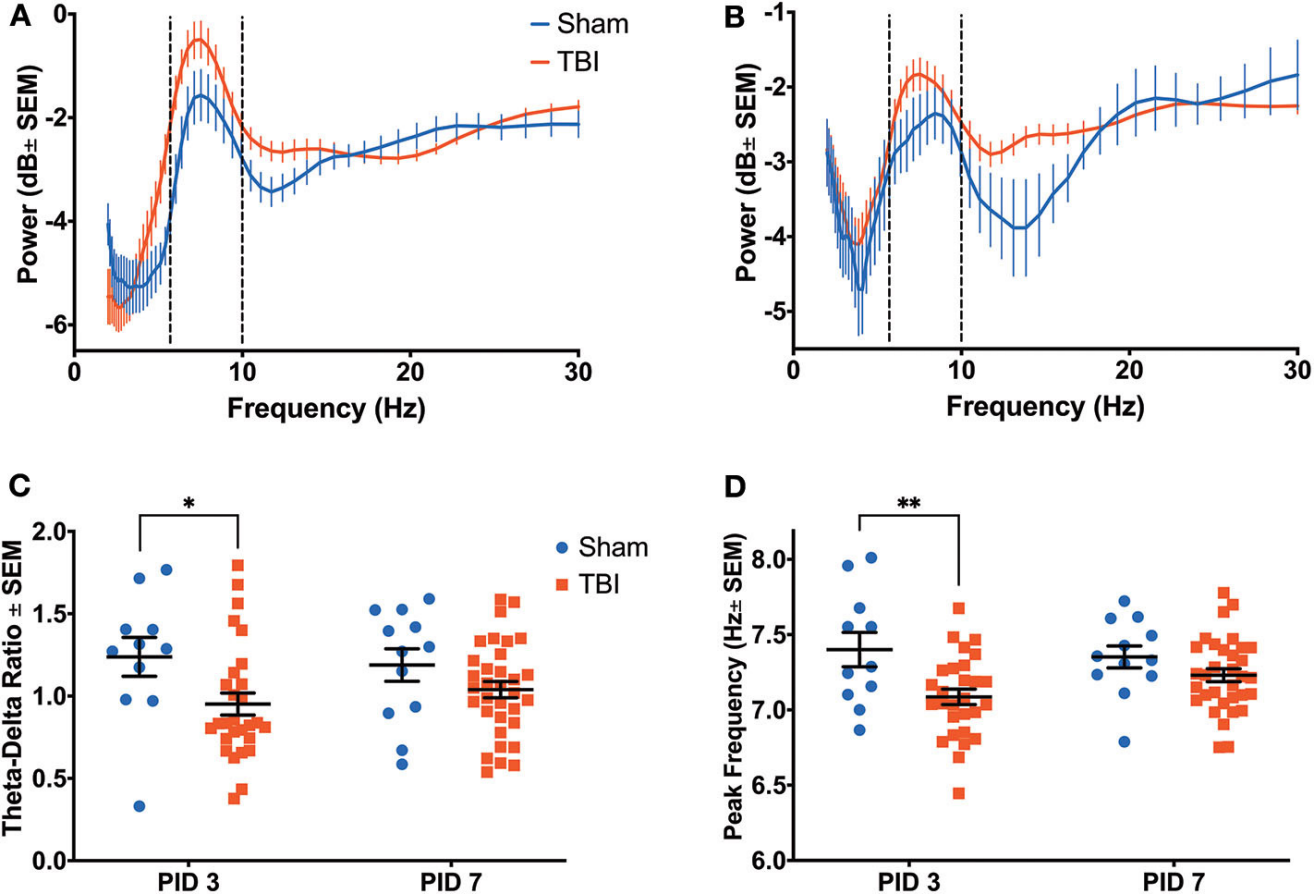
Anterior Cingulate Recovery Following Injury. A significant effect of power (*p* < 0.001), time (*p* < 0.001) and group (*p* < 0.001) on theta oscillatory power was detected by a three-way ANOVA. Dashed lines highlight the theta range (6–10 Hz). **(A)** A power spectral density from the ACC on PID3 reveals significantly higher theta power in injured animals than shams. **(B)** This difference persisted on PID7. **(C)** There was a main effect of group on TDR (*p* < 0.05), which was due to a significantly lower ratio in injured animals than sham animals on PID3. **(D)** There was a main effect of group on the theta frequency with peak power (*p* < 0.01), which was due to a significantly lower peak frequency in injured animals than shams on PID3. All data are presented as mean ± SEM, ^*^p < 0.05, ^**^p < 0.01. Adapted from Paxinos et al. (51).

Reductions in theta power were also observed in the ACC (Figures 5A,B), with significant effects of frequency [F_(9, 440)_ = 3.467, *p* < 0.001], time [F_(1, 360)_ = 48.29, *p* < 0.001], and group [F_(1, 360)_ = 39.35, *p* < 0.001], as well as a significant interaction between time and group [F_(9, 360)_ = 7.095, *p* < 0.001]. Similarly to the dHPC, there was an apparent alteration in theta power on PID 3 (Figure 5A; Sham = −2.1405 ± 0.4538 dB; TBI = −1.1033 ± 0.3173 dB); yet unlike in the dHPC, differences in average theta power between Sham (−2.5972 ± 0.3768 dB) and TBI (−2.0710 ± 0.2135 dB) groups appear to persist on PID 7 (Figure 5B).

While there was no effect of group, there was main effect of time on delta power [F_(1, 1, 040)_ = 117.2, *p* < 0.001], as well as an interaction between time and group [F_(1, 1, 040)_ = 4.734, *p* < 0.05]. However, there was a primary effect of group [F_(1, 44)_ = 6.242, *p* < 0.05] on TDR (Figure 5C), which was due to a significantly lower ratio in injured animals on PID3 (Sham = 1.238 ± 0.118; TBI = 0.952 ± 0.067; *p* < 0.05). Again, this value had recovered to sham-like levels by PID 7 (Sham = 1.189 ± 0.099; TBI = 1.039 ± 0.049; *p* = 0.27). Unlike in the dHPC, we found a main effect of group [F_(1, 80)_ = 10.43, *p* < 0.01] in peak theta frequency over time (Figure 5D). Injured animals had significantly slower peak frequency (7.086 ± 0.051 Hz) in the ACC than shams (7.400 ± 0.114 Hz) on PID3 (*p* < 0.01), but peak frequency in TBI animals (7.230 ± 0.043 Hz) had returned to sham-like values (7.352 ± 0.073 Hz) by PID7 (*p* = 0.39).

**Figure 6:**
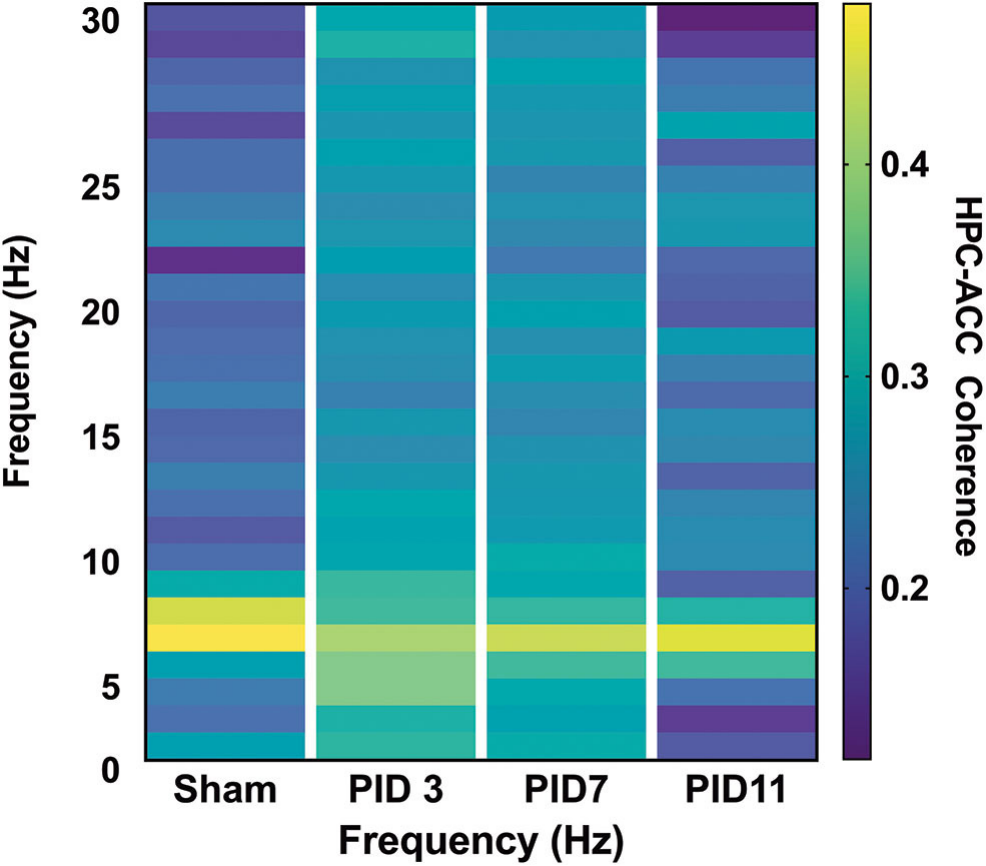
HPC-ACC Phase Coherence. Dashed lines represent the theta band (6–10 Hz). Adapted from Paxinos et al. (51).

Theta coherence between the top-powered hippocampal electrode and the ACC was evident in sham animals (Figure 6; median = 0.5447) but was significantly depressed in TBI animals on PID3 (Figure 6; median = 0.4053; *p* < 0.05) and PID7 (Figure 6; Sham median = 0.6577; TBI median = 0.4702; *p* < 0.001). By PID11, HPC-ACC coherence returned to sham-like levels (Figure 6; Sham median = 0.7133; TBI median = 0.6577).

### Effects of Injury and Stimulation on Spatial Learning

**Figure 7:**
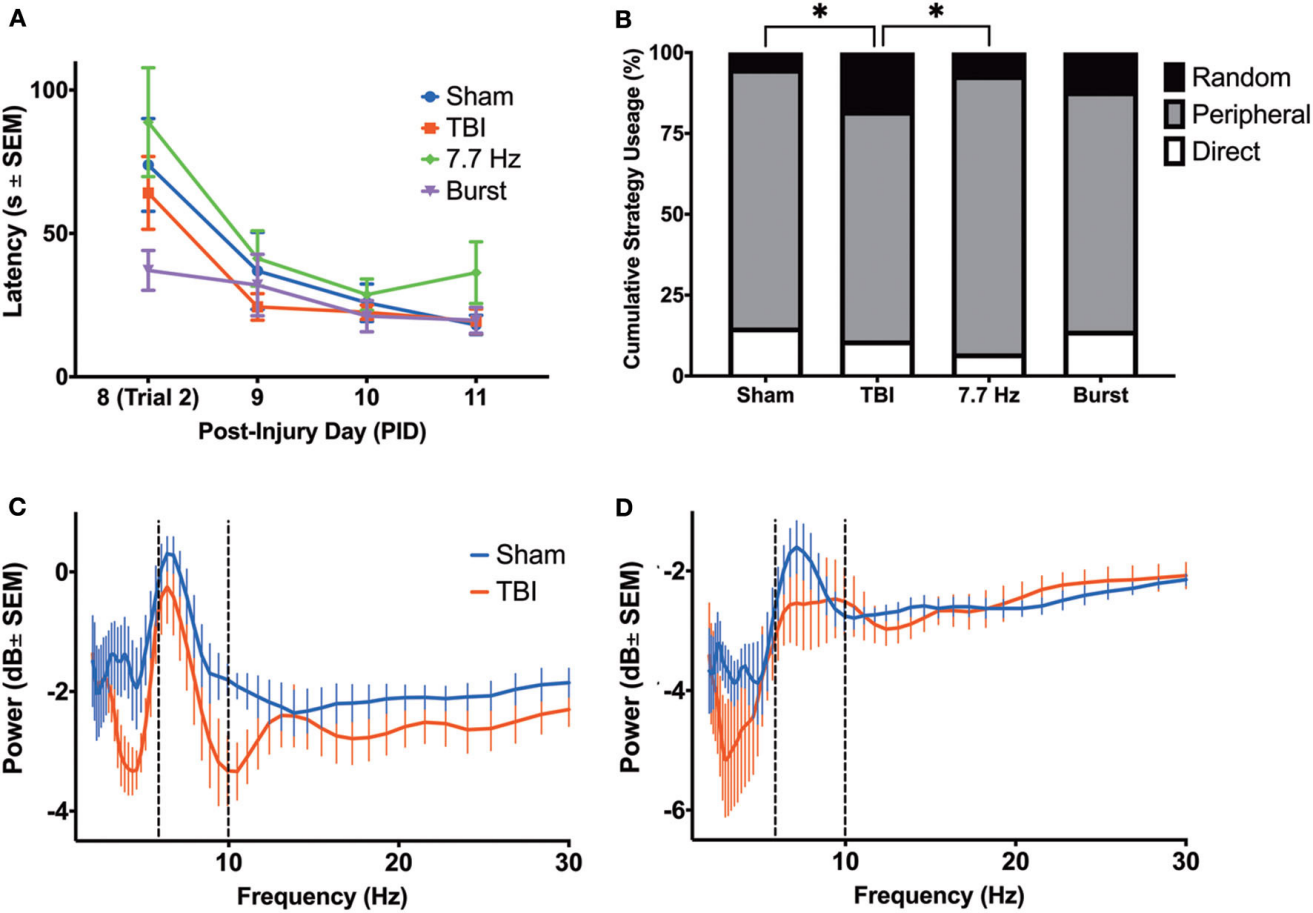
Barnes Maze Performance did not reveal a Robust Cognitive Deficit in Injured Rats. **(A)** There were no differences between groups in latency to complete the task; **(B)** however, there were significant differences in cumulative search strategy. Specifically, TBI animals used a random strategy significantly more often than shams (*p* < 0.05) and 7.7 Hz-stimulated animals (*p* < 0.05). **(C)** There were no differences in the theta power between groups in the hippocampus or **(D)** ACC on PID 11, prior to final day of maze trials. All data are presented as mean ± SEM, ^*^p < 0.05. Adapted from Paxinos et al. (51).

Beginning on PID8, animals underwent training on the Barnes maze. We analyzed latency (Figure 7A) and search strategy (Figure 7B) on trials 2–8. There was a main effect of day post-injury [F_(1.757, 63.83)_ = 21.43, *p* < 0.001] but no effect of group [F_(3, 37)_ = 1.519; *p* = 0.23] on latency to find the escape box. Subtle deficits in the search strategy were detected among the groups as TBI animals employed significantly different search strategies than sham [χ^2^(2) = 8.500, *p* < 0.05] and animals receiving continuous 7.7 Hz stimulation [χ^2^(2) = 7.162, *p* < 0.05]. There were no differences between groups in the number of errors committed (data not shown; F_(4, 40)_ = 0.6367; *p* = 0.64). Our initial hypothesis had been that persistent changes in theta oscillations would be predictive of spatial learning deficits. However, by PID7, it appeared that most measures of oscillatory activity were no longer different from sham. In addition, we analyzed hippocampal and cingulate LFP of sham and unstimulated injured animals before they were placed on the maze on PID11 to determine whether, after several days of behavioral testing, oscillations remained similar between sham and injured animals. In fact, multiple *t-*tests (with a Bonferroni correction for multiple comparisons) found no significant effect of group on theta or delta power in the HPC (Figure 7C) or the ACC (Figure 7D), nor significant differences on PID11 TDR (data not shown), supporting the conclusion that theta oscillations had largely normalized by the end of Barnes maze training.

## Discussion

Invasive electrophysiology provides a window into local neural activity, particularly as it pertains to specific behaviors such as spatial learning. We and others have demonstrated in rat and mouse models of TBI that oscillatory activity in the dHPC is diminished following a moderate injury (43, 46–48). Of particular relevance, theta oscillations, which have a well-described role in plasticity and spatial learning (42, 57–59), were attenuated. In the current study, acute LFP were recorded (3 and 7 days) after lateral fluid percussion injury in a novel context. Animals were then evaluated for performance on the Barnes maze spatial learning task, with power assessed again on the final day of training. We observed attenuation of theta power in the HPC and a trend toward a change in delta power. Moreover, there was a significant decrease in TDR but no change in MPF. In the ACC, theta power was increased, and there was not a significant change in delta power. In addition, there were significant changes in both TDR and MPF in the ACC. Finally, there was a significant increase in beta phase coherence detected between two hippocampal electrodes and a decrease in theta coherence between the HPC and ACC. However, unlike in our previous studies, most of the changes were observed only on post-injury day 3 and had resolved by 7 days post-injury. All measures from TBI animals were statistically similar to sham by day 11. Critically, when we evaluated spatial learning on the Barnes maze on days 8–11, we did not detect a difference in escape latency between injured rats and sham controls. Only a subtle but significant deficit in learning strategy was observed between sham and TBI rats, which is attributed to a greater percentage of random searches by injured animals. In summary, we observed a recovery of oscillations over the first week of injury and that injured animals with improved oscillations performed similarly to sham animals on the Barnes maze as evaluated by escape latency.

### Theta Oscillations and TBI

Depolarization in the dendrites (sources) and the hyperpolarized soma (sinks) result in individual neurons generating electric fields, creating a dipole. As pyramidal neurons and their projections are well-organized in layers, local ionic activity can sum, creating LFPs [*reviewed in* Buzsáki et al. (60)]. In cases of little neural activity, or if activity is truly random, the resulting trace on an EEG or in a LFP would be flat. However, coordinated neural activity results in regular swings (oscillations) in electrophysiology traces and phase relationships between oscillations in distal, communicating brain regions (61, 62). In rodents, slow wave theta oscillations are associated with cellular plasticity. Specifically, long-term potentiation (LTP) is greatest when high frequency stimulation (HFS) is timed to the peak of the theta oscillations (58). Moreover, multiple HFS with an inter-stimulation interval in the theta frequency range (theta burst) results in a greater potentiation as compared to a single HFS (63). When rodents are exposed to a novel environment, navigate a familiar environment, or perform either spatial or object-based tasks, robust theta oscillations can be recorded in the dHPC (42, 64). When oscillations are attenuated chemically (41, 42), or following brain injuries such as TBI or epilepsy (43, 44, 47), cognitive performance declines. Critically, theta oscillations have been detected in patients undergoing invasive monitoring during the performance of spatial learning and recall tasks (31, 32, 62, 65, 66). In these patients, the presence (or absence) of theta is a good predictor of performance on non-spatial recall and retrieval tasks (30, 45, 67).

Previously, our lab demonstrated that, following a moderate fluid percussion injury, theta frequency oscillations were disrupted over the first week following injury, corresponding with significant increases in latency to find the platform and suboptimal search strategies on the Barnes maze (43, 47). These findings were in line with previous chemical lesion studies in which inactivation of the medial septum led to disrupted hippocampal theta oscillations and impaired performance on the radial arm maze (68) and Morris water maze (53). Similar to our previous observations, Paterno et al. (48) found a decrease in hippocampal theta power concomitant with cognitive dysfunction following lateral fluid percussion injury in a mouse. However, they not only observed attenuated theta but also a general broadband reduction of oscillations across multiple frequency bands. Therefore, they could not contribute their behavioral findings strictly to alterations in theta. Looking specifically at individual neurons, Munyon et al. (69) identified a change in bursting properties of hippocampal neurons that correlated with impaired performance on the novel object task. While findings from each of these three groups were somewhat different, they similarly agreed that disruptions in hippocampal electrophysiology following TBI resulted in impaired cognitive performance. In line with these studies, we now demonstrate that injury not only alters oscillations in the hippocampus but also in the ACC. Moreover, coherence between neighboring regions in the hippocampus, as well as between the hippocampus and ACC, are disrupted post-injury. Unlike these previous studies, we observed a recovery in oscillatory activity that coincided with a lack of a behavioral deficit. One region from which we have yet to record in our laboratory is the peri-contusional cortex. In fact, others have now reported that pathological high frequency oscillations between 100–600 Hz in this cortical region correlate with the eventual development of spontaneous seizures (70, 71). Ultimately, it is clear that changes at the subcellular and cellular level following TBI lead to alterations in network properties, and that changes at the systems level correspond with altered functional outcomes.

In addition to oscillatory activity at a single contact, we also evaluated the phase coherence of oscillations between two dHPC electrodes as well as between the dHPC and the ACC. Hippocampo-cortical projections are important for coordinating oscillatory activity across distal brain regions during cognitive tasks (62). We hypothesized that any changes in intracortical communication would predict dysfunction in spatial learning. In fact, some groups have reported hyperconnectivity following TBI, while others have demonstrated reductions (72–76). Injury-related atrophy disconnects brain regions, leading to neurologic dysfunction (77, 78). Therefore, it is hypothesized that the combination of damage to some key areas and compensation by other, abnormal areas results in a mixed pattern of hyper- and hypoconnectivity. In fact, we observed increased intrahippocampal coherence in the beta range but decreased theta coherence between the hippocampus and ACC on PID3 and 7. Importantly, all of the abnormalities showed a trend toward recovery on PID7 and a full return to sham-like levels by PID11.

### Vulnerability, Metabolism, and Oscillations

One of the many unanswered yet critically important questions related to TBI is when an individual might be ready to return to play or work without vulnerability to a second insult. Moreover, is it possible to develop an objective measure of recovery that is non-invasive, easily deployed and interpreted, and also cost-effective? One hypothesis is that, due to mitochondrial and metabolic dysfunction following injury (79–87), the brain would be unable to respond to a second insult and therefore be vulnerable. There is a well described body of literature that demonstrates a period of metabolic dysfunction following TBI (80–82, 88), and that even a more mild TBI can reduce glucose uptake for several days to weeks in patients (89). Moreover, in rodent models, the period of metabolic dysfunction corresponds to times of vulnerability (85, 87, 90–93). Interventions to increase metabolic function result in improved outcome, as measured by neuronal survival (94, 95) and cognitive improvements (96, 97). One of the key findings in this paper is that, following injury, animals initially lost weight, were lethargic, and had depressed neural activity, as measured by attenuated theta oscillatory activity. However, following a similar time course as recovery of metabolic function (88), oscillatory activity and cognitive function improved across the dHPC and ACC. We hypothesize that a decrease in metabolic activity necessarily results in diminished neural activity and therefore altered oscillatory activity in the LFP.

As recently described by Agoston et al. (98), it is critical to consider biologic measures and diagnostic tools that can be used across species if we hope to successfully translate our findings from the lab to the clinic. The authors suggest neuroimaging and blood-based proteomics as two techniques that can be used to follow disease progression longitudinally, regardless of species (98). We now propose that electrophysiology represents an affordable and easily deployable tool in the laboratory, the playing field, or the clinic that can provide longitudinal and objective data related to disease progression and recovery of function following TBI. Recently, connectivity has gained interest as a potential biomarker for injury. Connectivity can be analyzed during various cognitive tasks using fMRI (74, 75), scalp EEG with graph theory analysis (73, 99, 100), or via coherence analysis of LFP. Patient studies have linked altered connectivity with severity of cognitive decline and extent of anatomical damage (74). A connectivity analysis of scalp EEG in a juvenile porcine model of mild rotational TBI found similar changes in oscillatory activity. Specifically, decreases in delta (1–3.5 Hz), theta (4–7.5 Hz), and alpha (8–12 Hz) power were observed on PID4 and 7, as well as a dramatic, progressive increase in hyperconnectivity between the 32 scalp electrodes (99).

The ability to quantify similar oscillations from scalp EEG as from depth electrodes raises the diagnostic potential of oscillations. In fact, clinical studies have demonstrated some potential of EEG for determining injury severity based on other oscillatory abnormalities (101, 102). Evaluations of EEG as a prognostic indicator have also been favorable. In particular, EEG phase and coherence were determined, via discriminant analysis and multivariate regression analyses, to be some of the best predictors of functional recovery outcome after closed head injury (103). If similar signals can be detected from EEG and LFP, as suggested in these porcine and patient data, then EEG represents an affordable, easily deployed, and non-invasive technique to longitudinally track injury progression and/or recovery of function across a range of species from rodent to porcine to primate. Due to the heterogeneity in TBI, it will be important for future studies to incorporate a wider range of injury severities in order to address the predictive value of this tool in the context of a disorder with high inter-individual variability.

### Biomarkers to Determine Injury Severity and Recovery of Function

As described above, Agoston et al. (98) have suggested that neuroimaging and blood-based proteomics can be used to follow disease progression longitudinally regardless of species. Traditionally imaging (104, 105) and blood biomarkers (106, 107) have been used as diagnostic and prognostic tools in patients who have experienced a TBI. Recent studies have evaluated the potential of these tools to follow recovery longitudinally in rodent models. Using a rodent model of mild (108) and repeat mild (109) fluid percussion injury, Wright et al. (108) investigated used MRI and blood based biomarkers to track injury over a 1 month period. Specifically, following a single mTBI, injured rats had only a transient cognitive deficit, recovering within a week following injury (108). In these animals, no macroscopic brain injury was observed with a ${\text{T}}_{2}^{*}$-weighted MRI; however, transient changes in magnetic resonance spectroscopy (MRS), diffuse tensor imaging (DTI), and protein biomarkers had largely recovered within 7 days post-injury. Persistent reductions in average streamline curvature detected in track-weighted imaging (TWI) were observed out to 30 days following injury. Animals receiving repeated mTBI had a different profile. Animals had persistent behavioral deficits, including in spatial learning and on the beam walk (109). Unlike animals receiving a single injury, there was evidence of structural brain damage using ${\text{T}}_{2}^{*}$, and lasting changes in DTI, TWI, and blood biomarkers. Following a more severe fluid percussion injury, MRS was able to detect longitudinal changes in neurochemical profiles, including those related to metabolic status, edema, excitotoxicity, oxidative stress, and inflammation (110). Similar to our findings, others have demonstrated that EEG has the potential to track injury progression and recovery of function in a clinically-relevant porcine models of rotational TBI (99, 111, 112). Therefore, there is a growing volume of preclinical data establishing the potential of non-invasive biomarkers including neuroimaging, blood-biomarkers, and electrophysiology to assess acute injury, track recovery of function, and/or evaluate vulnerability to second insult. Perhaps future studies could compare these markers in clinically relevant rodent and porcine models to determine whether similar patterns are observed across species and whether there is a difference in sensitivity between techniques. Ultimately, sensitive and objective tools to assess recovery following injury have the potential to aid clinicians and patient's families in determining when individuals have sufficiently recovered to return to work, play, or the field of military combat.

### Oscillations, Ambulation, and Stimulation

Based on our previous experience, animals can be lethargic in the immediate days following lateral fluid percussion. It is also known that oscillatory power can be driven by velocity, with higher speeds associated with higher theta power. In fact, we previously reported that changes in oscillatory power following moderate injury were at least partially explained by different amounts of activity and ambulation (47). In the current study, animals were initially recorded in a small novel context with insufficient room to run. It is still possible in this context that theta power was influenced, at least in part, by total movement; however, due to the lack of time-locked video, we were unable to measure total distance traveled to ascertain a relationship. It is clear that future studies of oscillations in the context of injury need to closely consider total movement duration, speed, and even acceleration when assessing changes in oscillatory power and coherence.

### Mild or Moderate Injury

Our original goal in this study was to address whether moderate lateral fluid percussion altered oscillations concomitantly in the hippocampus and ACC by determining if there was a change in phase coherence between these regions and to evaluate whether septal theta stimulation could improve both the electrophysiological and behavioral outcomes. While we used a drop angle that previously resulted in persistent cognitive deficits (50, 113), rats in this study did not develop a lasting cognitive phenotype, as determined by Barnes maze latency. Only a small but significant deficit in search strategy was detected. Additionally, unlike in our previous studies (43, 47), aberrations in theta oscillations had largely resolved within the first week post-injury. It is possible that a lack of effect is related to an extended duration of anesthesia necessary to implant the electrodes; however, this was not the case in our previous studies of electrophysiology following TBIs that resulted in a more persistent spatial learning deficit (43, 47). Regardless, we were unable to assess whether stimulation could have a beneficial effect on outcome, either in terms of oscillatory activity or latency on the Barnes maze. However, one interesting finding related to stimulation was that injured animals performed no worse or better with either fixed 7.7 Hz or theta burst stimulation. This is in contrast to our previous observation that septal theta stimulation tended to make spatial learning worse in sham animals (52, 55).

A key takeaway from these findings was recovery of oscillations in the first days following injury was associated with development of a mild cognitive phenotype. Specifically, as theta power returned to baseline, and coherence improved with time, rats exhibited only a small but significant change in search strategy to find the escape box. However, based on the drop angle used, the mortality observed, and the weight loss recorded, we chose not to describe our injury as mild, instead focusing on the phenotype. Recent reviews have highlighted the complexities of modeling mTBI, and the necessity of using clinically relevant models (114, 115). To test the hypothesis that electrophysiology can be used as a biomarker for injury severity, EEG and LFP must be compared across multiple injury severities and injury. Specifically, it is important that future studies consider the relationship of theta oscillations to outcome in specific clinically relevant models of mTBI, such as the closed head impact model of engineered rotational acceleration [CHIMERA; (116)], models employing a helmet (117), awake closed head injury (ACHI) models (118–120), and other models of more diffuse (121, 122) or focal injury (123). For a biomarker to have translational relevance, it should be able to identify changes related to initial injury severity, as well as the associated behavioral phenotype.

### Future Studies Must Address Sex and Age

While this study was funded prior to the update of the NIH guidelines related to sex as a critical biological variable, it is clear that future studies evaluating the relationship of oscillations to cognitive function will need to include rats from both sexes. Moreover, as TBI is more likely to occur in children and in the elderly (3, 124), it is also critical to evaluate age as a relevant biological variable when considering the potential of any biomarker.

## Conclusions

There is mounting evidence that changes in neural activity following TBI can be tracked using electrophysiology. Previously, we and others have demonstrated in rodent models of TBI that altered hippocampal oscillations are observed concomitantly with deficits in learning. In the current study, oscillatory indices had largely recovered by the end of the first week post-injury, and unlike in those previous studies, substantial behavioral deficits were not observed. In addition to these prior findings, we now demonstrate that oscillations are disrupted not only in the hippocampus but also the ACC. Moreover, connectivity is disrupted both within the hippocampus and between the hippocampus and ACC, as measured by theta phase coherence. If similar changes in specific electrophysiological signatures, such as theta power or TDR, can be detected with scalp EEG, then electrophysiology represents a potentially affordable, rapid, and easily deployable way to measure recovery of neural activity following TBI. Non-invasive, quantifiable, and objective measures of neural activity have the potential to provide patient and doctor with important information about an individual's recovery, whether that be related to diminished vulnerability and the ability to return to play, or improved activity and plasticity and the potential to return to a job or classroom setting. Future studies are needed to continuing evaluating the clinical potential of this tool in a wider range of injury severities, ages, and sexes. There is a clear need to continue to investigate network level changes in electrophysiology in the context of TBI to better understand the nature of both chronic disability and recovery of function.

## Data Availability Statement

The raw data supporting the conclusions of this article will be made available by the authors, without undue reservation.

## Ethics Statement

The animal study was reviewed and approved by University of California Davis Institutional Animal Care and Use Committee.

## Author Contributions

KO re-analyzed all of the data, generated the final figures, and prepared the manuscript as submitted. AP and KT participated in study design, collected data, and generated initial analyses and figures. AS, AI, AE, and SC provided support related to Matlab code development, data analyses, and statistics. KS played a role in the development of the hypotheses, study design, and framing the context of the data. GG was involved in all aspects of the study. All authors contributed to the article and approved the submitted version.

## Conflict of Interest

The authors declare that the research was conducted in the absence of any commercial or financial relationships that could be construed as a potential conflict of interest.
